# Intraoperative Fluid Management in Patients Undergoing Spine Surgery: A Narrative Review

**DOI:** 10.3389/fsurg.2020.00045

**Published:** 2020-07-29

**Authors:** Corinna Ongaigui, Juan Fiorda-Diaz, Olufunke Dada, Ana Mavarez-Martinez, Marco Echeverria-Villalobos, Sergio D. Bergese

**Affiliations:** ^1^Department of Anesthesiology, The Ohio State University Wexner Medical Center, Columbus, OH, United States; ^2^Department of Anesthesiology, School of Medicine, Stony Brook University, Health Sciences Center, Stony Brook, NY, United States

**Keywords:** spine surgery, prone position, fluid therapy, fluid management, perioperative care

## Abstract

Fluid management has been widely recognized as an important component of the perioperative care in patients undergoing major procedures including spine surgeries. Patient- and surgery-related factors such as age, length of the surgery, massive intraoperative blood loss, and prone positioning, may impact the intraoperative administration of fluids. In addition, the type of fluid administered may also affect post-operative outcomes. Published literature describing intraoperative fluid management in patients undergoing major spine surgeries is limited and remains controversial. Therefore, we reviewed current literature on intraoperative fluid management and its association with post-operative complications in spine surgery.

## Introduction

During the last decade, the role of fluid therapy as an essential component of perioperative management has been given more emphasis in large part due to the Enhanced Recovery After Surgery pathway (1, 2). The impact of intraoperative fluid therapy on hemodynamics during major surgery has been widely studied (3, 4). However, established guidelines for intraoperative fluid therapies are limited, particularly in specific surgical populations such as patients undergoing major spine surgery.

Fluid management during major spine surgery is determined by procedure- (e.g., length of surgery, risk of increased intraoperative blood loss, and prone positioning) and patient-related factors (e.g., American Society of Anesthesiologists –ASA physical status, comorbidities, and sequelae from long-term spinal deformities) (5–8). Over the past decade, the number of elective spine surgeries has continuously increased, especially in surgical populations at high risk of perioperative complications such as the elderly. A reduced physiological reserve is one of the factors that has a significant impact on the response to intraoperative fluid therapy in this patient setting (9). Likewise, the volume and type of fluid administered during the surgery may be associated with the onset of post-operative complications (10–15).

The spectrum of complex or major spine surgery comprises correction of deformities (e.g., scoliosis), vertebral infection (e.g., abscess), and spinal stabilization procedures following trauma, neoplastic disease or degenerative changes. Major spinal surgery may be performed at any spinal segment, from cervical to lumbosacral, and involves multiple vertebral levels.

Open and decompressive laminotomies and discectomies, anterior cervical discectomy and fusion, posterior cervical laminectomy and fusion, posterior cervical laminoplasty, multilevel anterior vertebrectomies, and fusion with instrumentation, decompressive laminectomies with posterior instrumented fusions, combined anterior/posterior approaches, and adult spinal deformity surgeries are some of the major spine procedures discussed in this review.

The use of spinal instrumentation devices may result in prolonged surgical times and significant intraoperative blood loss (16). Decompressive and corrective procedures involve multiple osteotomies releasing affected structures at localized spine levels. Spinal stabilization procedures entail instrumentation above and below the unstable vertebral levels in a combined posterior and anterior approach. Moreover, acute perioperative blood loss is a widely known complication of multilevel spine surgeries and is commonly exacerbated by the number of levels fused, osteotomies performed, and in pedicle subtraction osteotomies or vertebral column resections (17, 18). Blood transfusion in the setting of acute blood loss has been associated with increased risk of coagulation impairment, bloodborne infectious disease transmission, post-operative hematoma formation, shock, and pulmonary edema (16, 19).

Prone positioning is widely used in patients undergoing major spine surgeries and has been associated with significant hemodynamic changes such as decreased cardiac output, stroke volume (SV), and heart rate (20–22). Intraoperative hemodynamic stability and adequate tissue perfusion remain as some of the main challenges anesthesiologists face. Several trials suggest that goal-directed fluid therapy (GDFT) can help guide fluid administration to optimize intravascular volume status of patients undergoing major surgery. This hemodynamic strategy utilizes titration of fluids, vasopressors, and inotropes to achieve target values of hemodynamic variables tailored to the patient's cardiovascular physiology to improve post-operative outcomes (20, 23, 24).

### Objectives

In this narrative review, we intend to discuss published literature describing outcomes and correlation between intraoperative fluid management and post-operative complications (e.g., perioperative visual loss –POVL, delayed post-operative extubation, post-operative urinary retention –POUR, post-operative pulmonary complications, and prolonged intensive care unit –ICU and hospital length of stay –LOS) in patients undergoing spine surgeries. Pathophysiological changes associated with surgical positioning, the use of GDFT, and volume replacement during massive blood loss in spine surgery will also be addressed.

## Physiologic Changes During Prone Positioning

**Figure 1:**
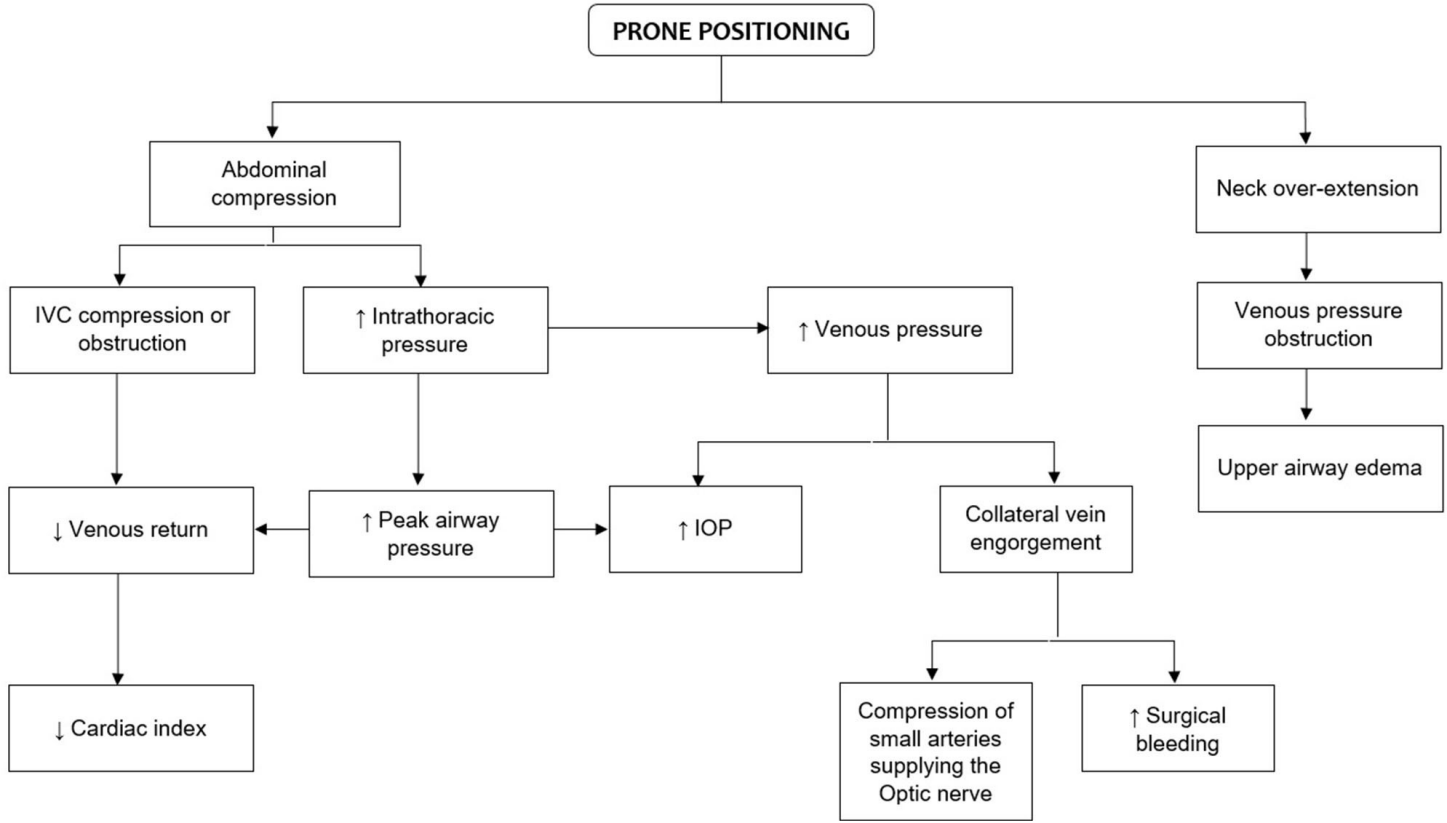
Physiologic changes of prone positioning that are relevant to spine surgery. IVC, inferior vena cava; IOP, intraocular pressure.

With the use of tools such as pulmonary arterial catheter and transesophageal echocardiography (TEE), significant reductions in cardiac output (CO) and SV have been reported in patients undergoing lumbar spine surgery in prone position (20, 21, 25). In an early study, Yokoyama et al. (21) analyzed several hemodynamic variables in anesthetized patients undergoing lumbar spine surgery in the prone position. Changes in systemic arterial pressure, right arterial pressure, pulmonary arterial pressure, CO, heart rate (HR), mean arterial pressure, pulmonary artery mean pressure, cardiac index (CI), stroke volume index (SVI), systemic vascular resistance index (SVRI), and pulmonary vascular resistance index (PVRI) were assessed. Authors reported a significant reduction in CI from 3.1 ± 0.5 to 2.5 ± 0.3 (*p* < 0.01) during prone positioning but no changes were observed in other hemodynamic variables (21). In healthy patients, these hemodynamic variations *per se* may have a minimum or null cardiopulmonary impact. However, some mechanical changes such as abdominal compression with subsequent increase in intrathoracic pressures also occur during prone positioning. Therefore, this may have a substantial impact on important anatomic structures (e.g., inferior vena cava), resulting in significant circulatory changes (i.e., decreased venous return, increased impedance of blood flow to the left ventricle and obstruction of the IVC) (22, 26). In addition, a greater intra-abdominal pressure generated during prone position may increase surgical bleeding by engorging collateral veins, compromising the spinal cord perfusion which can lead to lead to spinal cord ischemia (27–29). Likewise, changes in peak airway pressure and respiratory compliance can also contribute to decreasing venous return and CO (30) (Figure 1).

### Surgical Tables

The main goals of surgical positioning in patients undergoing spine procedures are to decrease venous pressures at the surgical levels and to reduce the burden on peripheral nerves and face while establishing an optimal surgical approach (31). In a randomized clinical trial, Dharmavaram et al. (20) sought to compare the hemodynamic effects between five different prone positioning variants (a bolster system, Siemens'—George and Tom Upholstery-, Andrews', Wilson's, and Jackson's frames) in patients undergoing lumbar surgery. Left ventricular areas, CO, CI, SV, and contractility score were some of the TEE variables measured in these patients. A significant decrease in CO was reported in all tested variants of which the Wilson frame position showed the greatest reduction (19%). Similarly, considerable changes in CI and SV were observed when the Andrews, Wilson, and Siemens systems were used (20). Nevertheless, the incidence of procedure-inherent complications such as acute kidney injury may be comparable regardless of the frame used (32). NICOM^®^ and FloTrac™/Vigileo™ systems are some of the non-invasive and minimally invasive (respectively), alternative techniques used to monitor hemodynamic changes and fluid response in patients undergoing spine surgeries in different prone variants (33, 34).

### Goal-Directed Fluid Therapy

The combined physiologic effects of prone positioning and the risk of substantial blood loss pose patients undergoing multilevel spine surgery at high risk for intraoperative hemodynamic instability. GDFT is described as an approach to guide perioperative fluid administration in order to avoid fluid overload, organ hypoperfusion, hypoxia, and hypovolemia (35, 36). This volume replacement strategy has been associated with more positive post-operative outcomes such as enhanced post-operative recovery and reduced length of hospital stay, occurrence of acute renal injury and infections (37–44). On the other hand, fluid overload has been linked to delayed post-operative extubation, perioperative visual loss, and increased length of hospital stay in patients undergoing spine surgery (45–52).

Published literature describing the use of GDFT in spine surgeries is limited (6, 13, 53, 54). Dynamic indices such as pulse pressure variation, stroke volume variation, corrected flow time, and plethysmography index variability have been commonly used to predict fluid responsiveness among GDFT trials (Table 1) (33, 34, 55, 56). Significant reduction in blood loss and blood transfusion, fewer post-operative respiratory complications, faster return of bowel function after surgery, shorter length of stay, reduced number, and duration of hypotensive episodes, and decreased incidence of nausea, vomiting and post-operative delirium have been reported with the use of GDFT in patients undergoing spine surgeries (6, 13, 53). The last finding is attributed to a reduction of peripheral inflammatory markers IL-6 and S100β and becomes of particular interest as fluid balance has been described as a modifiable risk factor of post-operative delirium in this patient setting (14). Additionally, GDFT plays an important role in optimizing intraocular pressure during prone positioning when compared to other fluid therapies or medications (e.g., α_2_ agonist Brimonidine) (54).

**Table 1:** Studies on monitoring fluid responsiveness during spine surgery in prone position.

**Author (year of publication)**	**Study design**	**Number of Patients, *N***	**Surgery**	**Key Findings/Points**
Biais et al. (46)	Prospective observational study	30	Scoliosis surgery	Prone positioning induced significant increase in PPV and SVV from the supine position, but their ability to predict fluid responsiveness remained unchanged.
Yang et al. (47)	Prospective observational study	44	Posterior lumbar spine fusion	PPV and FTc showed high fluid responsiveness predictability, with PPV proving to have a better predictability than FTc.
Kim et al. (48)	Prospective observational study	53	Posterior lumbar interbody fusion	Both PPV and PVI can be used to predict fluid responsiveness in patients undergoing spine surgery in the prone position using the Jackson table.
Min et al. (49)	Prospective observational study	40	Spine surgery in the prone position	The non-invasive NICOM^®^-derived SVV can be used to reliably predict fluid responsiveness in the prone position.

*PPV, pulse pressure variation; SVV, stroke volume variation; FTc, corrected flow time; PVI, plethysmography variability index*.

## Fluid Replacement During Massive Blood Loss

Despite an enormous amount of evidence supporting the substantial risk of greater perioperative blood loss during major spine and orthopedic surgeries, there is no universal consensus as to the optimal management of intraoperative fluid replacement for these procedures (18, 57, 58). In spine surgery, stripping muscles off bone and osteotomies are associated with an increased bleeding of exposed surfaces (17). Multi-level spine surgeries involve greater trauma and exposure, which means bleeding can be more insidious and profuse. Research efforts addressing massive perioperative blood loss in spine surgery are ongoing including preventive measures to reduce blood loss (e.g., tranexamic acid) and point-of-care devices (e.g., rotational thromboelastometry) that can be used to assess transient coagulopathies and therefore, to guide hemostatic therapy (59–62).

Crystalloids are the first-line resuscitative fluid in surgical patients (63–65). However, concerns about its overuse have been raised and studied (66–68). The high chloride content of 0.9% saline has led to hyperchloremic acidosis and increased risk of acute kidney injury (69–71). In addition, 0.9% saline use has been associated with more post-operative infections and blood transfusions compared to balanced salt solution in patients undergoing open abdominal surgery (72). This finding is consistent with *in vitro* and pre-clinical studies that show an association between hyperchloremic acidosis and changes in the immune response due to modifications in cytokine expression (73). Moreover, saline-induced acute kidney injury may be associated with neutrophil dysfunction and decreased bacterial clearance (74). In contrast, colloids are commonly preferred over crystalloids during massive bleeding based on their ability to persist longer within the intravascular space, enabling hemodynamic stability with a lower total volume of fluids (63). Nevertheless, colloids are costly and have been associated with hypersensitivity reactions, coagulopathy, and kidney dysfunction (75). Emerging evidence suggest that third-generation colloids in balanced salt solution are potentially safer with no significant effect on coagulation when compared to crystalloids (76, 77).

Intraoperative hypovolemia and vasodilation may result in hypotension and subsequent spinal cord ischemia. A decreased amplitude and increased latency in transcranial motor and somatosensory evoked potentials (TcMEPs and SSEPs, respectively) are some of the electrophysiological changes reported during spine ischemic episodes. In hypovolemic-hypotensive animal models, these electrophysiological variations return back to baseline values after blood volume was restored. However, the length of the hypotensive shock episodes remains as one of the main variables with a significant impact on these outcomes (78). Likewise, Lieberman et al. (79) also reported an association between hemorrhagic hypotension and vasodilatory hypotension with decreased amplitude and increased latency of TcMEPs in pig models. Nevertheless, reestablishment of the mean arterial pressure (MAP) during hemorrhagic hypotension (i.e., use of phenylephrine or preload optimization with colloids), did not restore TcMEP parameters as effectively as in the vasodilatory group (79). These findings suggest that augmentation of cardiac output and oxygen delivery (DO_2_) is more efficacious at restoring electrophysiological state after hemorrhagic hypotension than solely increasing MAP. A recent retrospective study conducted by Acharya et al. (80) including 61 patients who underwent spine deformity surgery with TcMEPs monitoring, identified hypotension as the most common cause of TcMEP's alterations. These findings were timely correlated with spinal cord ischemia as hypotension correction resulted in reversal of TcMEPs changes (80). While the authors followed a protocol to increase MAP > 100 mmHg, they did not specify how this was achieved.

## Fluid Management and Postoperative Complications in Spine Surgery

### Perioperative Visual Loss

Perioperative visual loss (POVL) in spine surgery is rare; however, it is one of the most devastating complications leading to greater functional disability and general medical burden (81). According to a nationwide population-based database, the incidence of POVL in spine fusion varied between 0.89 to 1.02 events per 10,000 surgeries during the period 1998–2012 (81). In another study that analyzed 167, 972 spinal deformity cases from 2009 to 2012, post-operative visual complication rate was 12.5 events per 100,000, with an average of 0.01% per year (82).

Ischemic optic neuropathy (ION), retinal arterial occlusion and cortical blindness are the most commonly reported causes of POVL (83), with patients undergoing spine surgeries involving thoracic or lower vertebrae fusions at higher risk (84).

A proposed pathophysiology for POVL in spine surgery suggests prone positioning may cause extravasation of fluids with subsequent interstitial fluid accumulation and elevated intraocular pressure. This elicits a local compartment syndrome within the optic nerve resulting in acute axon damage (85, 86). In addition, placing the patient in the prone position increases venous pressures resulting in venous engorgement. This in turn provokes a critical decrease in blood flow to the optic nerve by compressing the small supplying arteries, the posterior segment becoming especially vulnerable to ischemia due to poor collateral flow (49).

Several risk factors have been attributed to the development of POVL (83, 87) with volume and/or type of fluid administered being one of the foremost causes (48, 88, 89). In a case-control study of visual loss after cardiac surgery, a significant post-operative weight gain was commonly identified among patients, suggesting that large volumes of fluid may predispose a patient to visual complications (90). According to the ASA POVL Registry, mean intravenous crystalloid replacement was 9.7 ± 4.71 L among cases with 30% of them receiving colloids for fluid replacement therapy (84). Moreover, cell saver and packed red blood cells (PRBC) were used in approximately half of the cases (84).

In an attempt to reduce edema formation and to increase perfusion pressure of the optic nerve, several recommendations advocate the preferential use of colloids for intraoperative fluid replacement which traditionally have been taught to remain longer intravascularly due to their molecular size (49, 90–92). However, the general perception stating three times less colloid as crystalloid would be required to achieve similar hemodynamic effects in hypovolemic patients has become obsolete with this ratio being as low as 1:1.4-1.6 (93–95). Moreover, colloid administration may have little or no effect on IOP during prolonged spine surgeries in prone position. In a factorial randomized trial, Farag et al. (54) reported similar IOP in 60 patients undergoing spine surgery in the prone position receiving either crystalloid (lactated ringer) or colloid (5% albumin). Authors randomized patients into 4 groups: 2 of them receiving albumin and the other 2 receiving crystalloid, each combined with either an ophthalmic topical eye drop or topical Brimonidine, respectively. A significant increase in IOP was observed during prone positioning (mean of 12 ± 6 mmHg) in studied patients. This effect was significantly reduced with Brimonidine (*P* = 0.023) but neither the use of crystalloid nor colloid had any effect on IOP (*P* = 0.34) (54).

The correlation between the degree of blood loss, hypotension, and fluid therapy with ION remains controversial as these and other surgical-related risk factors have been linked to the development of POVL (96–98). Restricted intraoperative fluid therapies in the setting of large blood loss may result in anemia and hypotension. Anemia reduces oxygen carrying capacity and delivery to neural tissue whereas hypotension (i.e., hypovolemia or deliberate intraoperative hypotension) decreases arterial perfusion pressure, both potentially resulting to perioperative ION (97, 99). However, anemia and hypotension are common in many surgical procedures in which the incidence of POVL is basically null arguing further against their exclusive role in the pathogenesis of POVL (85). In a multivariate analysis, the POVL Study Group found that anemia or hypotension (defined as blood pressure > 40% below baseline for 30 min) alone were not independent risk factors of POVL, encouraging researchers to determine whether the effect of these factors remain significant when other variables (e.g., volume administration) are analyzed (49). Even though there is evidence supporting the administration of colloid over crystalloid to prevent POVL (10, 86, 100, 101), several safety and efficacy concerns about its use have been also reported (102, 103). In contrast, some authors support that either type of fluid can be used as long as adequate volume replacement is maintained and fluid overload is avoided (87, 104, 105).

### Delayed Post-operative Extubation

Perioperative fluid overload may prolong the time it takes for airway to return to baseline and delay post-operative extubation in patients undergoing major spine surgeries, especially when multiple levels are involved (45, 46, 106–108). Prone positioning and large fluid shifts can also result in facial and upper airway edema potentially precluding safe extubation (45–47, 109). Delayed extubation may occur in up to 44% of adult patients undergoing multilevel spine surgeries and in 20–25% of those involving thoracic or lumbar levels (45–47, 110). Likewise, the overall incidence of airway adverse events including re-intubation, failed extubation in the ICU or delayed extubation (i.e., inability to extubate the patient at emergence from anesthesia) have been reported to be as high as 27% after posterior occipito-cervical spinal fusion (111).

The administration of high volumes of IV fluids, regardless of the type, is commonly associated with an increased risk of post-operative respiratory complications (51, 112). In a retrospective study, Siemionow et al. (51) analyzed data from 1,307 patients undergoing spine surgery in order to assess the impact of fluid therapy on length of stay and pulmonary complications. After a multivariable logistic regression, a significant correlation between total fluids administered (>4,165 mL) and longer hospital stay was identified (*P* < 0.0001). This correlation remained significant when either crystalloids or colloids were administered (51). Moreover, Hart et al. (12) reported a lower incidence of prolonged post-operative intubation or reintubation among patients who underwent cervical spine surgery in the prone position after the implementation of a restricted fluid therapy protocol.

Similarly, prolonged duration of anesthesia and surgery, increased levels of vertebrae fused, and higher intraoperative blood loss and transfusions have been also associated with delayed extubation in patients undergoing spine surgeries (45–47, 109, 111, 113). A recent propensity score-matched analysis indicated that patients who received higher volumes of non-cell saver blood product transfusions and did not have a proportionate decrease in the volume of crystalloids administered. These patients were identified to be at risk for delayed extubation even after controlling for patient demographics, comorbidities, and complexity of the spine surgery (106). Research has shown that since most of the transfused blood products remain intravascular, there should be a reciprocal reduction of crystalloid fluid administration. Therefore, in the setting of increased intraoperative blood loss, immediate post-operative extubation may be feasible when the proportion of crystalloids administered is reduced based on the total fluids infused.

### Post-operative Urinary Retention

Postoperative urinary retention (POUR) is defined as an acute inability to void when the bladder is full after surgery, leading to patient's anxiety, discomfort, urinary tract infections, delayed hospital discharge and/or increased readmission rates (114–119). Most cases of POUR are associated with surgeries performed on the lumbar spine (15, 116, 119–121). Lumbar spine surgeries commonly involve the use of a Foley catheter which may cause urethral irritation and edema or reactive increase in tone of the bladder outlet, thereby contributing to POUR (120). In addition, post-operative pain in the lower lumbar area may trigger a reflex involving afferent and efferent fibers that ultimately inhibit detrusor muscle activity causing urinary retention (120, 122).

In the setting of posterior lumbar spine surgery, patient-related rather than surgery-related factors are likely to be the main contributors in the development of POUR (117, 119). Even though surgical manipulation of spinal nerves may be associated with the onset of POUR, no significant differences in POUR occurrence were reported between patients with and without overt post-operative neurologic deficit (119).

Despite some reports supporting a correlation between the volumes of IV fluid administered during surgery and the incidence of POUR in patients undergoing major surgeries (122–125), this association has not been observed in patients undergoing spine surgeries (15, 111). In a retrospective study including 397 patients who had undergone elective cervical, thoracic and lumbar surgery, Altschul et al. (116) reported an increased incidence of POUR in those patients with longer operative times. However, no correlation between POUR occurrence and intraoperative IV fluid administration was found (116). Similarly, Aiyer et al. (15) reported that a higher IV fluid volume administered was not an independent risk factor among patients who developed POUR after posterior lumbar spine surgery without intraoperative urinary catheterization. In summary, intraoperative bladder catheterization, previous spine surgery, operative level (L_2−5_), lumbar fusion, and duration of indwelling Foley catheterization are considered the main risk factors for POUR after major spine surgery (120).

### Prolonged Length of Stay

In a retrospective study, Nahtomi-Shick et al. (52) identified total fluid administration as one of the intraoperative factors with a significant impact on length of ICU stay in patients who underwent spine surgery. Greater volumes of crystalloid fluids administered during surgery were associated with longer ICU length of stay. However, total fluid administration is closely related to the type of procedure which limits its specific predictive value for ICU length of stay (52). In contrast, functional presurgical and post-surgical variables may be stronger predictors of length of stay in patients undergoing spine surgery (126). These variables tend to be heterogeneous and interrelated, making it difficult to determine each of their individual impacts on prolonging length of hospital or ICU stay (126, 127). For instance, complex procedures or higher ASA physical status scores are commonly associated with increased length of surgery with subsequent increased fluid administration (52, 127, 128). Likewise, longer surgery time has been associated with increased blood loss and transfusion; both of which are significantly correlated with longer hospital stay (128).

### Cerebrospinal Fluid Leak

Cerebrospinal fluid (CSF) leak is a well-known entity associated with a variety of neurosurgical procedures including spine surgeries. Whether planned or unplanned, CSF leak management may significantly impact patients' outcomes. Traditionally, specific surgical techniques such as the transoral approach for the anterior craniocervical junction pathologies, were linked to an increased risk of CSF leak and meningitis (129). However, the endoscopic endonasal approach has progressively replaced this and other highly invasive techniques for intra and extradural diseases, which resulted in enhanced recovery and a significant decreased risk of CSF leak (129).

Intentional or accidental durotomy triggers intracranial hypotension which, if uncorrected, may pose an increased risk of hemorrhage and ischemia. These life-threatening complications have been widely studied in patients undergoing intracranial procedures but may also be seen (in a lower proportion) after spine surgeries (130). In addition to surgical repair, optimizing intravenous fluid has been a traditional measure among practitioners (131). Nevertheless, specific guidelines on fluid replacement strategies and their impact on CSF leak resolution are limited.

### Other Complications

Deep vein thrombosis (DVT) and pulmonary embolism (PE) are life-threatening post-operative complications reported after spine and other neurosurgical procedures associated with prolonged surgical times (132). In addition, fluid and electrolyte imbalance have been identified as an independent risk factor for DVT and PE in patients undergoing lumbar spine surgeries (133). However, the impact of perioperative fluid management in the onset or prevention of DVT and PE in spine surgeries remains unclear. In a retrospective analysis of 207 patients undergoing cervical spine surgery, Dube et al. (134) noticed an incidence of pulmonary complications close to 40%. An univariate analysis showed a relative risk of pulmonary complications of 2.3 in patients who received > 2,000 mL of intravenous fluid when compared to those receiving ≤ 2,000 mL (*p* = 0.004) (134). Impaired breathing, pneumothorax, pleural effusion, acute respiratory distress syndrome and pneumonia were some of the pulmonary complications reported (134). Therefore, a fluid management strategy should be considered along with other interventions (such as the use of inferior vena cava filters), in patients at high risk of post-operative pulmonary complications after spine surgery (133, 134).

## Limitations

Considering the descriptive nature of our review, we intended to include relevant literature from several kinds of scientific journals. These highly variable sources of information allowed us to summarize different points of view describing the pathophysiology and outcomes associated with fluid management in adult patients undergoing spine surgeries. However, due to their particular complexity, we considered that including certain spine procedures (e.g., occipito-cervical junction and sacro-iliac surgeries) and populations (e.g., pregnant women, pediatric) in our narrative review, would have required a different approach from the one we initially proposed in order to better elucidate the heterogeneity of published literature.

## Conclusion

Perioperative fluid therapy in patients undergoing major spine surgeries represents one of the main challenges for anesthesia care providers. To our knowledge, no trials addressing fluid optimization strategies in spine surgery have been published. Prospective randomized clinical studies comparing different fluid therapy strategies are required in order to establish evidence-based guidelines for perioperative fluid management in patients undergoing major spine surgery. Future trials may also need to explore which methods of monitoring fluid responsiveness are most suitable to guide perioperative fluid administration in patients undergoing spine surgery in the prone position. Until more clinical evidence is collected, short- and long-term complications derived from intraoperative fluid overload can presumably be prevented by a better understanding of the physiologic changes in patients undergoing spine surgery.

## Author Contributions

CO performed literature search, worked on the structural design, and authored the manuscript. JF-D contributed to the structural design and critically revised the manuscript. OD and AM-M provided editorial advice. ME-V performed literature search, provided conception and structural design of the paper, and co-authored the main text. SB provided the publication concept and editorial advice. All authors reviewed the final manuscript before submission. All authors contributed to the article and approved the submitted version.

## Conflict of Interest

The authors declare that the research was conducted in the absence of any commercial or financial relationships that could be construed as a potential conflict of interest.
